# A Memoir on Vinous Fermentation

**Published:** 1804-05-01

**Authors:** 


### *A Memoir on Vinous Fermentation; by* CIT. THENARD.

THE vinous fermentation has hitherto received more attention than the acetous or putrid, not perhaps that it has any thing in it more remarkable, or more worthy of our consideration, but because it is in the natural order of things, to take more interest and set more value on what is of most immediate utility.

The date of the discovery of the vinous fermentation appears too well established to be called in question. All historians agree in saying, that the most ancient nations knew how to prepare spirituous drinks. It ascends therefore fore to the remotest times; and, if we pay any credit to the poets, we must carry it back to the fabulous ages. Indeed it would be surprising, if it escaped the notice of the earliest of men. An ebullition arising spontaneously in a liquid, a whole mass rising of itself, a sweet liquor becoming vinous, the change of a saccharine matter into an ardent spirit, are all extraordinary things, calculated to strike the attention, and awaken the desire of tracing them to their first causes. Accordingly there is no phenomenon more early observed, and none that has been the subject of more consideration, or has given birth to more experiments; yet, from one of those contrasts that rarely occur in the annals of science, though it has been the most studied, there is not perhaps one, with which we are less acquainted. It has been a rock, on which the endeavours of chemists, in all ages, have split. Beccher, so celebrated for his subterranean physics; Stahl, the Nestor of the ancient chemistry; Boerhaave, whose ideas were so great; Rouelle, to whom science is indebted for part of the progress it has made during the last half century; and Macquer, that master in the art of writing, all failed in their attempts to penetrate this mystery of Nature. Lavoisier, who was capable of surmounting the greatest obstacles, is the only person, who, enlightening the whole sphere of chemistry by his genius, travelled this obscure path without wandering out of his road. His enquiries into fermentation will ever remain a model of vegetable analysis. In this, as in every thing else he did, he observed that strictness of deduction, and accuracy of operation, which are his characteristics, and which may be considered as the source of the splendid discoveries, that will for ever illustrate his name. Still, notwithstanding the excellence of what he did, he was far from leaving nothing more to be done. Though he shed great light on this part of science, the obscurity with which it was enveloped was so great, that it is still seen only through the mist. This is a truth that did not escape himself; he was well aware that he had only laid open the path to the goal, which no doubt he would have reached, no doubt he would have completed the career he had begun with such success, had not death, jealous of his fortune and glory, robbed science of his labours.

All we know of fermentation in fact is confined to this, that the saccharine matter is converted into alcohol and carbonic acid by means of an intermediate substance. But what is the nature of this substance? and how does it act act on the sugar? These two grand questions form the subject of the present memoir; questions that have been often attempted, without ever having been solved. Some have thought, that the fermenting principle resided in the extractive matter. Others would have it to be in the mucilage, because this more frequently accompanies the extractive matter. Some, again deceived by the presence of tartar in wine, have imagined they found in this the true ferment; but if, instead of confining themselves to the fermentation of the must of grapes, they had turned their attention to that of other juices, in which analysis cannot discover the existence of this salt, they certainly would not have fallen into this error. Others, lastly, inconsiderately adopting all these opinions, have asserted, that a mixture of these different matters preside as it were over fermentation, effecting the decomposition of the sugar, and its conversion into alcohol.

Of these hypotheses, some are evidently false; others are seducing, and acquire from specious reasoning some degree of probability. But before we admit them, we must consult experience; we ought, leaving them all out of the question, to deduce from observation the theory, which we are too apt to form beforehand. If the genius of Stahl, instead of giving birth to phlogiston, a being that never existed but in the brilliant imagination of that extraordinary man, had interrogated nature by means of experiment more than he did, perhaps it would not have gone astray; perhaps Stahl would have discovered the truth, and deprived France of the glory of having produced the author of the modern theory of chemistry.

Such is the course I have pursued. Before I formed or adopted any system, I observed facts, and deduced from them consequences, which, it appeared to me, must guide us to the view of what passes in liquors under fermentation. But in a subject of such nicety nothing is more easy than to deceive ourselves; and it is particularly for the purpose of correcting my notions, if they be not just, that I submit to the world the result of my inquiries.

My first observations were made on the juice of gooseberries, which I had strong reasons to prefer to any other; its fermentation proceeding with most celerity, so that it is consequently best calculated to throw light on the causes that produce it. All my researches were directed at first to discover the matter, that serves as a ferment. It would be making a great step, and almost resolving the problem, or at least discovering a number of truths not yet yet known, to determine the nature of this matter, and to ascertain whether it be always one and the same, or whether there be several that possess this property. This important question had struck me long ago: I had even meditated upon it occasionally, and promised myself to attempt its solution, when the Institute proposed it as a subject of a prize. This was an additional strong motive to my pursuing it. I was far from being disposed to admit several fermentative principles; every thing led me to believe, that there was but one, and that it was none of those hitherto suspected, since in fact neither extractive matter, nor mucilage, nor tartar, &c. acts upon sugar. But this required positive demonstration; and though I have yet no absolute proofs of it, as it is by no means demonstrated that there are several, and one is observed on all occasions, this opinion seems to me at present to deserve the preference.

Through a linen cloth of close texture I pressed out the juice of a killogramme of gooseberries. It was turbid, and held in suspension a slightly glutinous matter, which I separated by the filter, and washed in a large quantity of water. As nothing is to be neglected in experimental science, and the most trifling fact frequently leads to important consequences, I subjected this matter to a regular examination. I first mixed it with sugar and water, to see whether it would cause them to ferment; and I soon perceived many bubbles of an elastic fluid to be extricated, which I found to be carbonic acid. The effervescence continued a week, and at the end of this period the liquor was a pleasant drink, but slightly saccharine; it contained a great deal of alcohol, and might easily have been mistaken for a wine not completely made. It may be supposed I redoubled my zeal and attention in the examination of a substance, that offered me what I sought. It was natural first to inquire, whether the whole of it were adapted to decompose sugar; but a sixth part of its weight being scarcely able to effect this decomposition, I concluded, that it contained the fermentative principle only in small quantity. This I attempted every method in vain to separate, and obtain apart: nothing therefore remained for me, but to compare it before and after it had served to produce fermentation. It was not apparently altered by this process; being still insipid, insoluble both in water and in alcohol, and affecting neither infusion of litmus nor syrup of violets; but on distillation it no longer afforded any trace of volatile alkali. This result, at which

I was

I was not surprised, and which a second experiment confirmed, was nevertheless a ray of light, that confirmed me in the course I should pursue. It showed me, that the germ of fermentation was of an animal nature, it agreed with the ideas I had before conceived, and gave to my suspicions an appearance of reality.

I now examined the juice of gooseberries with great care, to discover this animal matter, which I considered already as the true ferment. As it was insoluble by itself, it must be combined with some substance, that held it in solution. All the reagents I employed failing to answer the purpose, I had recourse to fermentation itself, and observed the phenomena produced by it under all circumstances. I made my experiments on nearly a litre of filtered and perfectly clear juice. The apparatus was placed in a stove, where the thermometer stood at 20°: it was not long before a fermentation was evident; a large quantity of carbonic acid was presently evolved; much froth was formed; the liquor lost its transparency, and it even became so turbid, that a sediment was thrown down, which was more evident as the fermentation approached its end. This sediment was of a yellowish white colour, glutinous, void of taste, grew brown on drying in the open air, and became slightly acid. Thrown on red-hot coals it burnt in the same manner as animal substances: distilled in a small retort it afforded a considerable quantity of carbonate of ammonia even crystallized. It made sugar ferment with extreme promptitude. In short, it was a substance perfectly analogous to the yeast of beer.

I was eager to try whether this phenomenon were general, as it ought to be according to my mode of reasoning: and in fact experience soon taught me, that it was common to all juice in a state of fermentation. The must of grapes, the juice of cherries, pears, peaches, and apples, and the decoction of barley and of wheat, afford yeasts in their fermentation. The grape juice yielded more than the others, though less than that of gooseberries: accordingly it did not ferment so readily as the latter. The juices of cherries and peaches deposited nearly the same quantity: those of pears and apples afforded very little, which is the reason why their fermentation is so slow. I could have wished to have had a greater number of fruits at my disposal, that I might have varied my experiments more: they were sufficient, however, to prove, that, where alcohol is formed, a sediment of yeast is commonly formed likewise. If they who have have any doubt on the subject remaining will maturely consider the two following experiments, I believe they will find themselves convinced. I knew that honey diluted with water would gradually be converted into a liquor containing spirits. Cullen informs us, that the saccharine urine of a diabetic patient undergoes in time the same change. Accordingly I set both of these to ferment, and the sediment of yeast was formed in each.

It may be laid down, therefore, as demonstrated, that in every spirituous fermentation an animal matter is deposited, similar in all respects to that arising from wort, possessing absolutely the same properties, and in particular that of decomposing sugar, and converting it into carbonic acid and spirit of wine. This gives rise to a new question, that naturally presents itself, and ought next to occupy our attention. Is the yeast generated in the process of fermentation, or rather was it already formed, and did serve as a ferment?

I must confess we have yet no experiments, which directly prove, that Nature employs this substance exclusively to effect the conversion of sugar into alcohol and carbonic acid. For why should it be deposited when the fermentation has taken place? It may be said indeed, that the sugar holds it in solution, that it can dissolve more than is necessary for its decomposition, and that then the excess is precipitated. But this theory is feebly supported by experiment. Is this a sufficient reason to reject it altogether? Have we not several instances of compounds, that require much time for their formation? And this perhaps is what occurs in the juices of fruits, where the ferment and the sugar are long in contact with each other. What is certain, or what at least appears probable, is, that if yeast be a product of fermentation, as all liquors that ferment deposit it, no doubt it owes its origin to one and the same soluble substance, from which it differs little, and which produces it by its reaction on the sugar.

Whichever of these two opinions obtains the preference, after more numerous trials, as I have no doubt that yeast is an immediate principle of vegetables, and in consequence acts an important part in the phenomena both of Art and Nature; as I am likewise persuaded, that, if any other substance capable of exciting fermentation exist, it is in the highest degree analogous to yeast; that it differs from it very little; that it is composed, like it, of azot, oxygen, carbon, and hydrogen; and lastly, that it has, no no doubt the same mode of acting on sugar: I shall proceed to exhibit with the greatest care the properties of this matter, which I shall henceforward term ferment, and in particular consider well its action on the saccharine principle, in order to establish the theory of fermentation. This theory, even supposing yeast not to exist already formed, but to be produced in fermenting juices, will still be of utility, and capable of various applications, as will appear below.

I shall not recapitulate the physical qualities of ferment, as I have mentioned them several times already; but I shall confine myself to its chemical properties, which alone are of essential import. It has no taste. It neither reddens infusion of litmus, nor changes syrup of violets green. The putrid fermentation, which in time it undergoes, is in every respect similar to that of animal substances. By desiccation it loses three-fourths of its weight, and this loss consists entirely of water. Thus dried it is still capable of exciting fermentation; it is by no means decomposed, and may be preserved in this state without alteration for an indefinite time. We may avail ourselves of this property, to convey it to places at a distance from any brewery, or with which it is so difficult to keep up an intercourse, that fresh yeast could not be sent to them, particularly in summer, without becoming putrid. Distilled in a small retort, and urging the fire so far as this would bear, eight parts of ferment left a residuum of 2.83 of coal; and I obtained 1.61 of water, 1.31 of oil, and 1.46 of muriat ammonia on adding muriatic acid. Finally, I collected 0.33 of gas, containing a fifth of its bulk of carbonic acid, and which, when this was separated from it by potash, burnt like carbonated hydrogen, and required 1.5 of oxygen for its combustion. From this experiment we see, that ferment contains in particular a great quantity of carbon.

Water at the temperature of 12° or 15° does not dissolve $\frac{1}{100}$ of ferment: indeed, it dissolves so little, that after standing upon it several hours, and being well filtered, it has scarcely any action upon sugar. Boiling water occasions it to undergo a decomposition, which I shall examine in another memoir.

Nitric acid, even diluted with water, at eighteen degrees, decomposes it also: it converts it into grease; and there is evolved from it at first azot mingled with carbonic acid, and afterward nitrous gas at the same time.

Potash acts upon this substance in the same manner as upon animal matters, and the phenomena in both cases are perfectly the same: with each it forms a saponaceous substance, and a great quantity of ammonia, that it volatilizes.

But of all the properties of ferment, no one is so remarkable, and at the same time so useful, consequently no one so much deserves to be studied, as its action upon sugar: this is interesting to men in every class of society, from the mechanic to the philosopher; to both by its products, and to the latter because it may be a fertile source of reflection and of new truths. It is much to be regretted, that Lavoisier did not pursue the investigation as he intended, and examine it with that care, which is conspicuous in all his labours. Who was more capable of giving birth to a theory of fermentation, than the author of the modern theory of chemisty? No doubt he was prevented from doing this by a concurrence of circumstances: and this theory, important as it is to science, has hitherto remained vague and hypothetical. Knowing the fermentative principle, it could not avoid naturally making a part of my researches: if I have not rendered it as clear as I hoped, at least the veil with which it was covered is removed, and it rests on reasoning confirmed by experiment.

To obtain the solution of this problem, I added together different quantities of ferment and sugar; I observed in every case what became of both; and I confirmed by farther observations what the preceding had suggested. Sixty grammes (927 grains, or nearly one ounce of ferment) not dried, and three hundred grammes (4630 grains) of sugar entered into fermentation readily, the temperature being 15° (centigrade=59° Fahrenheit.) In four or five days all the saccharine matter had disappeared; 51.5 litres (3041 cubic inches) of carbonic acid had been evolved; the liquor being filtered, and distilled to two-thirds, gave on a second rectification 863 grammes of spirit at 13°. The apparatus was so contrived, that nothing was lost: the receivers were cooled with common salt and ice. I found by synthesis, that this quantity of spirit was equivalent to 171.5 grammes of alcohol at 39°. The residues left after distilling the spirit were poured into dishes and evaporated to dryness: from the residuum of the second distillation nothing was obtained, but that of the first yielded about 12 grammes of a nauseous substance, slightly acid, and feebly attracting the moisture of the air. I wished to discover the nature of this acid, but there was too little to ascertain it. Lavoisier says, it is the acetous. Lastly, of the sixty grammes of ferment, there remained forty grammes mes of a substance, which I believed to be more animalized than the ferment itself. I was much surprised to find, that I obtained from it by distillation much less ammonia. Hence I suspected, that by mixing it afresh with sugar, fermentation would again take place, and thus all the azot would disappear. What I had foreseen occurred: at the end of seven days, having filtered the liquor, I obtained as a residuum thirty grammes of a substance, which on distillation gave no trace of volatile alkali. I was persuaded, that the azot was carried off with the carbonic acid gas. To convince myself of this, I collected near 41 litres of the carbonic acid in an inverted vessel filled with solution of caustic potash. The whole was absorbed, which leaves no doubt of its purity.

What then becomes of the azot? it ought to be found either in the residuum of the ferment, or in the residuum obtained by evaporating the liquor left after distillation, or in the alcohol: but the residuum constitutes only half the ferment employed; the quantity of matter left by 300 grammes of sugar and sixty of ferment amounts only to twelve grammes; and neither of these yields any ammonia on distillation, while ferment affords a great deal. If these observations be just, if I have accurately noticed all the phenomena, if nothing have misled me, we cannot avoid concluding, that the azot must exist in the alcohol. Yet I have sought in vain to discover its presence in this fluid, in ether, and in the acetous acid: on passing these through tubes heated red-hot in the fire, and burning the gasses in Volta's eudiometer by means of the electric spark, we obtain such small quantities, that they are by no means sufficient to decide the question: 24 or 25 parts of gas yield at most one of residuum.

I have made several other experiments however, which hitherto tend to show, that azot may exist in such a manner as not to be discovered on distillation, consequently that it may be one of the constituent principles of vegetables, though in general when distilled they afford no ammonia. But as I have not repeated these experiments, and they are of such importance, that we cannot be too reserved in announcing them, I purpose to revise and vary them; I will endeavour to appreciate all the circumstances, and if I obtain convincing proofs, I will not delay communicating them to the public.

However this may turn out, these results afford us sufficient light, to see what passes in the act of fermentation. In this respect I cannot agree in opinion with Lavoisier.

 I do

I do not believe as he does, that all the carbonic acid formed proceeds from the sugar. How, on such a supposition, can we conceive the ferment to act upon it? I think, that the first portions of acid are owing to a combination of the carbon, of the ferment, and the oxygen of the sugar, and that the ferment gives rise to fermentation by abstracting from the sugar a portion of this principle. To render this idea more clear, I suppose a particle of sugar to be formed of eight parts of oxigen, four of carbon, and one of hydrogen, which is not very remote from the truth, according to the experiments of Lavoisier: one of these eight parts of oxygen will unite with a fourth part of the carbon of ferment, and then, the equilibrium between the principles of the sugar being disturbed, they will combine in a different manner, so as to form carbonic acid and alcohol. The ferment has in fact a strong attraction for oxygen; as is proved by its decomposing air with the greatest facility; when acetous and carbonic acids are produced, and the azot is disengaged. If pure air be employed instead of common air, the re-action is still more speedy. I have introduced 15 grammes of ferment into a vessel filled with a litre of pure air; I opened it over quicksilver; a fifth of its bulk was absorbed; the ferment was grown sour, all the oxygen gas had evidently disappeared, and was converted into carbonic acid: the temperature was 15°.

From what has been said we see what becomes of the carbon of the ferment, and we shall learn what may become of its other principles, if we recollect the quantity of products resulting from a given quantity of matter subjected to fermentation, and comparing the nature of the one with the other.

From sixty grammes of ferment, and three hundred grammes of sugar we obtained, carbonic acid 95 grammes, pure alcohol 171.5; extractive matter, slightly acid, and containing no azot, 12; residuum of the ferment 40. These 40 grammes still contained 25 of ferment, so that 35 only had been employed for the decomposition of the sugar; and these 35 were reduced to 15 of a white substance insoluble in water, incapable of acting upon sugar, yielding no ammonia on distillation, and leaving a coal, that burns with scarcely any residuum; in short, exhibiting characters that distinguish it from all other substances, induce me to consider it as a peculiar matter.

It appears then, that ferment takes oxygen from sugar, not only by means of part of its carbon, but also by means of of part of its hydrogen; for the quantity of carbon given out by ferment is too small, to be the sole germ of fermentation. The azot disappears, and enters perhaps into the composition of the alcohol; the other principles of the ferment form acetous acid, and a peculiar insoluble white matter, which is precipitated. The acteous acid remains in solution in the liquor left after distillation, with an extractive matter, proceeding no doubt from the sugar, and foreign to it.'

It is not probable, that the elements of the sugar, in their re-action upon each other when the equilibrium between them is disturbed, form water: there is very little hydrogen in sugar, and a great deal in alcohol; and besides, on adding together the quantity of carbonic acid, of alcohol, of extractive matter, and of residuum, we have a deficiency of one eleventh only of the matter by which they were produced. This loss must be attributed to the water the sugar contains, and is by no means owing to alcohol being carried off by the carbonic acid. Of this I have been convinced myself by receiving more than thirty litres of this gas in caustic potash; and by distillation and rectification I obtained only a few grammes of fluid, which had so little taste of spirit, that it was not to be distinguished.*

This theory appears to me so much the more probable, because it perfectly accords with facts; and this truth becomes particularly striking when we compare them. I am far, however, from considering it as complete. Time no doubt will bestow on it the perfection it wants; and I hope myself soon to add to its evidence. I know not whether I shall be able to discover what becomes of the azot of the ferment: but I shall ascertain without difficulty, whether the residual matter obtained, which I consider

---

* Cit. Seguin has laid before the Institute a very different theory of fermentation. He thinks, that in this process water is decomposed, that its oxygen unites with the carbon of the ferment, and produces carbonic acid, while its hydrogen combines with the sugar, and converts it into alcohol. For this theory to be admissible, we should obtain more alcohol than there was sugar; but the fact is, little more than half its weight is produced, and besides, supposing all the carbon of the decomposed ferment to be converted into carbonic acid, at most not above a sixth part of the quantity actually produced would be formed, as we may easily convince ourselves from calculations already established. It may likewise be objected to this theory, that sugar contains a great deal of oxygen, and alcohol very little.

sider as a peculiar substance, be a product of fermentation as I believe; whether the sugar contribute to its formation which is possible; or whether it be ready formed, and merely precipitated, which is contrary to all probability. The experiments, that remove all my doubts on this head, I shall relate in a second Memoir, in which I shall not only elucidate such points in this as may appear equivocal, or at best resting on too slight foundations, but I shall also exhibit all the particulars that result from them. Here, on the contrary, I have avoided them as much as possible, and endeavoured to consider the phenomenon only in a general way.

